# Whole genome sequence of the *Treponema pallidum* subsp. *endemicum* strain Iraq B: A subpopulation of bejel treponemes contains full-length *tprF* and *tprG* genes similar to those present in *T. p.* subsp. *pertenue* strains

**DOI:** 10.1371/journal.pone.0230926

**Published:** 2020-04-01

**Authors:** Lenka Mikalová, Klára Janečková, Markéta Nováková, Michal Strouhal, Darina Čejková, Kristin N. Harper, David Šmajs

**Affiliations:** 1 Department of Biology, Faculty of Medicine, Masaryk University, Brno, Czech Republic; 2 Department of Immunology, Veterinary Research Institute, Brno, Czech Republic; 3 Department of Population Biology, Ecology, and Evolution, Emory University, Atlanta, Georgia, United States of America; University of Lincoln, UNITED KINGDOM

## Abstract

*Treponema pallidum* subsp. *endemicum* (TEN) is the causative agent of endemic syphilis (bejel). Until now, only a single TEN strain, Bosnia A, has been completely sequenced. The only other laboratory TEN strain available, Iraq B, was isolated in Iraq in 1951 by researchers from the US Centers for Disease Control and Prevention. In this study, the complete genome of the Iraq B strain was amplified as overlapping PCR products and sequenced using the pooled segment genome sequencing method and Illumina sequencing. Total average genome sequencing coverage reached 3469×, with a total genome size of 1,137,653 bp. Compared to the genome sequence of Bosnia A, a set of 37 single nucleotide differences, 4 indels, 2 differences in the number of tandem repetitions, and 18 differences in the length of homopolymeric regions were found in the Iraq B genome. Moreover, the *tprF* and *tprG* genes that were previously found deleted in the genome of the TEN Bosnia A strain (spanning 2.3 kb in length) were present in a subpopulation of TEN Iraq B and Bosnia A microbes, and their sequence was highly similar to those found in *T*. *p*. subsp. *pertenue* strains, which cause the disease yaws. The genome sequence of TEN Iraq B revealed close genetic relatedness between both available bejel-causing laboratory strains (i.e., Iraq B and Bosnia A) and also genetic variability within the bejel treponemes comparable to that found within yaws- or syphilis-causing strains. In addition, genetic relatedness to TPE strains was demonstrated by the sequence of the *tprF* and *tprG* genes found in subpopulations of both TEN Iraq B and Bosnia A. The loss of the *tprF* and *tprG* genes in most TEN microbes suggest that TEN genomes have been evolving via the loss of genomic regions, a phenomenon previously found among the treponemes causing both syphilis and rabbit syphilis.

## Introduction

The spirochete *Treponema pallidum* subsp. *endemicum* (TEN) causes endemic syphilis (bejel), a chronic infection usually localized to mucosal and skin lesions. Highly related human pathogenic treponemes include *T*. *pallidum* subsp. *pallidum* (TPA), which causes syphilis, and *T*. *pallidum* subsp. *pertenue* (TPE), which causes yaws. Although the TEN spirochete is highly related to the syphilis and yaws treponemes [1–3], there are differences in the geographical distribution, transmission routes, and clinical symptoms of bejel, yaws, and syphilis. Whereas bejel is found in drier climates, corresponding to the sites where the two reference laboratory strains were isolated (Bosnia, in Southern Europe, and Iraq, in Western Asia), yaws is found in warm, moist climates in Africa, Southeast Asia, and the western Pacific (reviewed in [4,5]). Bejel and yaws are usually transmitted by direct skin-to-skin or skin-to-mucosa/mucosa-to-skin contact with an infected person or by contact with contaminated utensils [4,6] and the contact is usually of a non-sexual nature, whereas syphilis is mainly venereally transmitted and can be transmitted from mother to child during pregnancy or during birth resulting in congenital syphilis. However, exceptions to these rules occur. Several imported cases of yaws and bejel in children and adults in Europe and North America have been described [7–10]. In addition, recent studies by Noda et al. [11] and Kawahata et al. [12] have identified TEN in clinical samples from Cuban and Japanese patients, respectively, who had previously been diagnosed with syphilis.

The differential diagnosis of bejel, yaws, and syphilis based on clinical symptoms is quite difficult, especially in the early stages of disease. Since serology cannot discriminate between infection caused by TEN, TPA, and TPE, epidemiological data together with clinical symptoms are the only tools with which to diagnose the infectious agent. Variability in clinical symptoms and transmission modes make precise identification of the causative agent in early-stage treponemal infection difficult, if not impossible [9]. The recent identification of TEN in Cuba and Japan among patients diagnosed with syphilis demonstrates the difficulties inherent to diagnosis based exclusively on serology, geographical occurrence, clinical symptoms, and anamnestic data [11,12] and emphasizes the role of molecular tools for differentiation between TPA/TPE/TEN infections. Bejel, classified so far as endemic treponematosis, might be more widespread than originally expected especially due to the recent findings of sexual transmission of this neglected disease and ability to mimic syphilis infection [9–12]. Thus, the real incidence or prevalence of bejel cases remains unknown. Identification of genomic sequences specific for treponemal subspecies would therefore help to improve diagnosis of different treponematoses [13].

To date, only a single TEN genome sequence, that of the lab strain Bosnia A, has been finished [2], hindering our ability to analyze the genetic diversity present within TEN strains. In this study, a high-quality complete genome sequence of the TEN strain Iraq B was assembled using a pooled segment genome sequencing (PSGS) technique and Illumina sequencing. To our knowledge, TEN Iraq B and TEN Bosnia A are the only known and available bejel-causing laboratory strains. As shown in this study, a subpopulation of TEN Iraq B treponemes contains the *tprF* and *tprG* loci that were previously described as deleted in the TEN Bosnia A genome [2].

## Materials and methods

### Amplification of TEN Iraq B DNA

Isolated DNA from TEN Iraq B came from the collection of Kristin N. Harper from the Department of Population Biology, Ecology, and Evolution, Emory University, Atlanta, Georgia, USA, who received the DNA from the US Centers for Disease Control and Prevention (CDC, Atlanta, USA). The DNA was isolated in CDC from an unknown serial rabbit passage of the TEN strain Iraq B. Originally, the Iraq B strain was isolated in 1951 in Iraq, from a 7-year-old girl who had oral mucous patches and anal condyloma [14], clinical evidence typical of bejel. The genomic DNA of the TEN Iraq B strain was first randomly amplified with the REPLI-g Single Cell kit (Qiagen, Hilden, Germany), according to the manufacturer's instructions. The randomly amplified DNA was further amplified with specific primers using the PSGS method as described previously [2,15–17]. Briefly, the DNA of the Iraq B strain was amplified by PrimeSTAR GXL DNA Polymerase (Takara Bio Inc., Otsu, Japan) as 285 separate overlapping amplicons (for primers see S1 Table). The PCR cycling conditions were set up as follows: initial denaturation at 94°C for 1 min; 8 cycles: 98°C for 10 s, 68°C for 15 s (annealing temperature gradually reduced by 1°C/every cycle), and 68°C for 6 min; 35 cycles: 98°C for 10 s, 61°C for 15 s, and 68°C for 6 min (43 cycles in total); followed by a final extension at 68°C for 7 min. Subsequently, PCR products were purified using a QIAquick PCR Purification Kit (QIAGEN, Valencia, CA, USA) and mixed in equimolar amounts into four distinct pools. These pools were separately sequenced using Illumina technology to separate sequencing of paralogous chromosomal regions.

### DNA sequencing and assembly of the Iraq B genome sequence

Whole genome DNA sequencing was performed using the Illumina NextSeq 500 next-gen sequencer (Illumina, San Diego, CA, USA). Sequencing reads were preprocessed using Trimmomatic v0.32 with the sliding window of 4 bp and average quality threshold value equal to 17. Minimal read length was set to 48 bp. The genome of Iraq B was assembled from both reference mapped reads and assembly of *de novo* contigs, assembled using IDBA_UD (v. 1.1.1; [18]). The TEN Bosnia A genome sequence was used as a reference (CP007548.1; [2]). Assembled individual reads or contigs were aligned to the TEN Bosnia A reference sequence using Lasergene software (DNASTAR, Madison, WI, USA). Finally, all gaps and discrepancies were individually resolved using Sanger sequencing of corresponding amplicons (n = 21). The repetitive regions of the *arp* (TENDIB_0433) and TENDIB_0470 genes were also amplified separately, and the corresponding PCR products were Sanger sequenced to obtain the precise number of repetitions in these genes. Altogether, 7 genomic regions (covering genes TENDIB_0040, TENDIB_0348, TENDIB_0461, TENDIB_0697, TENDIB_0859, TENDIB_0865, and TENDIB_0966) revealed intrastrain variability in the length of homopolymeric (G- or C-) stretches. These regions were amplified, and the prevailing length of these regions was determined by Sanger sequencing.

### Gene identification, annotation, and classification

The whole genome sequence of the Iraq B strain was assembled from Illumina contigs and Sanger sequencing reads. The Geneious software v5.6.5 [19] was used for gene annotation based on the annotation of the published TEN Bosnia A genome [2], and a gene size limit of 150 bp was applied. Iraq B genes were tagged with TENDIB_ prefix. The *tprK* gene (TENDIB_0897) contained intrastrain variable nucleotides, and the variable nucleotide positions were denoted with Ns in the complete Iraq B genome.

### Comparison of whole genome treponemal sequences

Whole genome alignment of treponemal sequences was constructed with Geneious [19] and SeqMan (DNASTAR, Madison, WI, USA) software using the following genomes: TEN Iraq B (CP032303.1), TEN Bosnia A (CP007548.1; [2]), TPE CDC-2 (CP002375.1; [16]), TPE Gauthier (CP002376.1; [16]), TPE Samoa D (CP002374.1; [16]), TPE Ghana-051 (CP020365.1; [20]), TPE CDC 2575 (CP020366.1; [20]), TPE LMNP-1 (CP021113.1; [13]), TPE Kampung Dalan K363 (CP024088.1; [21]), TPE Sei Geringging K403 (CP024089.1; [21]), TPE Fribourg-Blanc (CP003902.1; [17]), and TPA SS14 (CP004011.1; [22]). Complete genome sequences, except for chromosomal regions showing recombination (*tprK* sequences, tRNA-Ile and tRNA-Ala intergenic spacers of both rRNA operons, *tprD* gene) or containing repetitions (*arp*, and TP0470 genes), were used to construct a phylogenetic tree. There were a total of 1,129,405 positions in the final dataset. The phylogenetic tree topologies were inferred employing RAxML-NG (v0.9.0) using TN93 substitution model [23]. Gamma-distributed substitution rates among sites and proportion of invariable sites were applied. Trees were constructed using optimized topology, the branch lengths and rate parameters option with BIONJ tree used as a starting tree [24]. The robustness of the tree branches was assessed by 500-bootstrap replicate analyses. Predicted tree was visualized by iTOL (v5.5) [25].

### Identification of intrastrain heterogeneity

Genetic heterogeneity was identified according to the protocol described elsewhere [20,21,26,27]. Briefly, individual Illumina reads were mapped to the final version of the TEN Iraq B genome sequence using SeqMan NGen (v4.1.0) software with default parameters. Specifically, a 93% read identity relative to the reference sequence was required to align reads to the reference genome (i.e. TEN Iraq B). The haploid Bayesian method was used for SNP calculation using the SeqMan NGen (v4.1.0) software. Individual reads supporting a less frequent allele located at the 3’-terminus (i.e., five or less nucleotides) were omitted. Loci with genetic heterogeneity within the TEN Iraq B genome were defined as those with a minor allele frequency of 8% or more in regions having a coverage depth greater than 100x. All identified sites were then visually inspected using SeqMan NGen (v4.1.0) software. The *tprK* (TP0897) gene, which showed intrastrain variability, was omitted from the analysis.

### Nucleotide sequence accession number

The complete genome sequence of the Iraq B strain was deposited in GenBank under accession number CP032303.1.

## Results

### Whole genome sequencing, genome parameters, gene annotation

Whole genome sequencing of the TEN Iraq B strain using the PSGS technique revealed a total average coverage of 3469×. The length of the Iraq B genome (1,137,653 bp) was identical to the length of the Bosnia A genome, and there was an overall gene synteny between the TEN Iraq B and Bosnia A genomes. Both genomes contained the *tprD2* allele at the *tprD* locus [28] and had identical *rrn* spacer patterns (Ala/Ile in the first and second *rrn* operon, respectively) [29]. Altogether, 1125 genes were annotated in the TEN Iraq B genome, including 54 untranslated genes encoding rRNAs, tRNAs, and other ncRNAs. Differences in the number of pseudogenes were found between the TEN Iraq B and Bosnia A genomes. A summary of the genomic features of the two TEN strains is shown in Table 1.

**Table 1:** Summary of the genomic features of the TEN strains Iraq B and Bosnia A.

Genome parameter	TEN Iraq B	TEN Bosnia A
GenBank Accession No.	CP032303.1	CP007548.1
Genome size	1,137,653 bp	1,137,653 bp
G+C content	52.77%	52.77%
No. of predicted genes	1125 including 54 untranslated genes	1125 including 54 untranslated genes
Sum of the intergenic region length (% of the genome length)	52,289 bp (4.60%)	52,643 bp (4.63%)
Average/median gene length	978.8/831.0 bp	979.2/831.0 bp
No. of genes encoded on plus/minus DNA strand	600/525	600/525
No. of annotated pseudogenes	19*	15**
No. of tRNA loci	45	45
No. of rRNA loci	6 (2 operons)	6 (2 operons)
No. of ncRNAs	3	3

*Pseudogenes comprised those identified during comparison of the sequence of TEN Iraq B with TEN Bosnia A sequence (TP0146, TP0279, TP0461, TP0479, TP0520, TP0532, TP0812, TP0865, TP1029 and TP1031) and those identified during comparison with TPA Nichols and TPE Samoa D sequences (TP0082a, TP0129, TP0132, TP0135, TP0266, TP0318, TP0370, TP0671 and TP1030).

**Pseudogenes annotated in the sequence of TEN Bosnia A resulted either from comparison with TPE Samoa D sequence (TP0082a, TP0146, TP0316, TP0370, TP0520, TP0532, TP0812, TP1029) or with TPA Nichols sequence (TP0129, TP0132, TP0135, TP0266, TP0318, TP0671 and TP1030).

### The overall genome structure of TEN Iraq B

Despite the identical size of the Iraq B and of the Bosnia A genomes (1,137,653 bp), several genomic differences were found. The genomes differed in the number of repetitions in the TP0433 and TP0470 genes. Whereas the TEN Iraq B genome contained ten 60-bp long repetitions in the *arp* gene (TP0433), the TEN Bosnia A genome harbored only eight such repetitions. On the other hand, the number of 24-bp long repetitions in TP0470 was higher in the Bosnia A genome (n = 20) than in the Iraq B genome (n = 15). Most of the differences between both genomes were single nucleotide variants (SNVs) including substitutions (n = 37; Table 2) and indels (n = 4; altogether covering 7 nucleotides). The indels were found in a short homopolymeric region (501274–501278; coordinates according to TEN Iraq B) in which Iraq B contained a 5C sequence whereas Bosnia A contained a 4C sequence, and in 3 additional regions containing 2-nt long repetitions (295333–295344 containing 6 AG repetitions in Iraq B, whereas Bosnia A contained 5 such repetitions; 370688–370691 containing 2 GT repetitions in Iraq B, whereas Bosnia A contained 3 such repetitions; 1123140–1123149 containing 5 CG repetitions in Iraq B, whereas Bosnia A contained 4 such repetitions). In addition to substitutions and indels, differences between the genomes were also found in the length of 18 homopolymeric tracts (S2 Table), defined as stretches of identical nucleotide sequences longer than 7 nucleotides.

**Table 2:** Genetic differences between TEN Bosnia A and Iraq B genomes.

TEN Iraq B (CP032303.1) coordinate	Nucleotide in TEN Iraq B	Nucleotide in TEN Bosnia A	Gene	Gene coordinates	Protein	Amino acid replacement(Iraq B/Bosnia A)
18409	G	A	TP0017	1	hypothetical protein	M/M
18413	T	C		5		V/A
80362	T	C	TP0073	260	HDOD domain protein	K/R
135228	G	T	TP0117	1451	TprC	P/E*
135229	G	C		1450		P/E*
135246	C	T		1433		R/K
136528	C	T		151		E/K
136542	C	T		137		R/H
188037	G	T	TP0165	477	TroC	L/L
203271	G	A	TP0186	368	HemN	G/E
230525	C	A	TP0225	241	hypothetical protein	P/T
320541	A	G	TP0304	1580	hypothetical protein	M/T
330101	G	A	TP0313	1042	TprE	V/I
333269	C	T	TP0316	801	TprG	G/G
372199	T	C	IGR TP0347-8**	-	-	-
450049	G	A	TP0422	769	hypothetical protein	A/T
468671	C	T	TP0442	1298	RecN	A/V
497702	G	A	TP0470	75	hypothetical protein	L/L
512065	G	C	TP0483	1187	hypothetical protein	S/W
521209	A	G	TP0488	819	Mcp2-1	R/R
521388	T	C		998		F/S
534764	G	A	TP0500	952	penicillin-binding protein	A/T
566812	T	G	TP0524	5	S16 family endopeptidase La	E/A
592250	C	T	TP0548	1043	FadL-like protein***	P/L
643057	G	A	TP0592	744	hypothetical protein	M/I
690733	G	A	TP0632	929	TprS	R/H
702522	G	C	TP0641a	94	hypothetical protein	L/V
725892	A	G	TP0664	46	FlaA	T/A
728789	A	C	TP0667	546	bifunctional phosphoribulokinase/uridine kinase	H/Q
882333	A	G	TP0814	69	TrxB	A/A
942845	G	A	TP0864	67	hypothetical protein	L/L
993147	T	G	TP0915	1030	hypothetical protein	F/V
994934	T	C	TP0917	1320	MgtE	F/F
1032779	C	T****	TP0952	620	putative lipase/esterase	G/E
1085945	T	C	TP0999	261	FtsK	A/A
1089703	C	T	TP1001	552	hypothetical protein	A/A
1130225	A	G	TP1035	731	ValS	L/P

Listed are 37 single nucleotide variants. Differences in the number of repetitive sequences and differences in homopolymeric regions are not listed. The hypervariable *tprK* (TP0897) gene was excluded from the analysis.

*P/E replacement is a result of two adjacent nucleotide changes.

**IGR, intergenic region.

***Protein predictions by Radolf and Kumar [30].

****A region comprising 7 T nucleotides.

### Similarity of the Iraq B genome to the available TEN Bosnia A and TPE genomes

**Fig 1:**
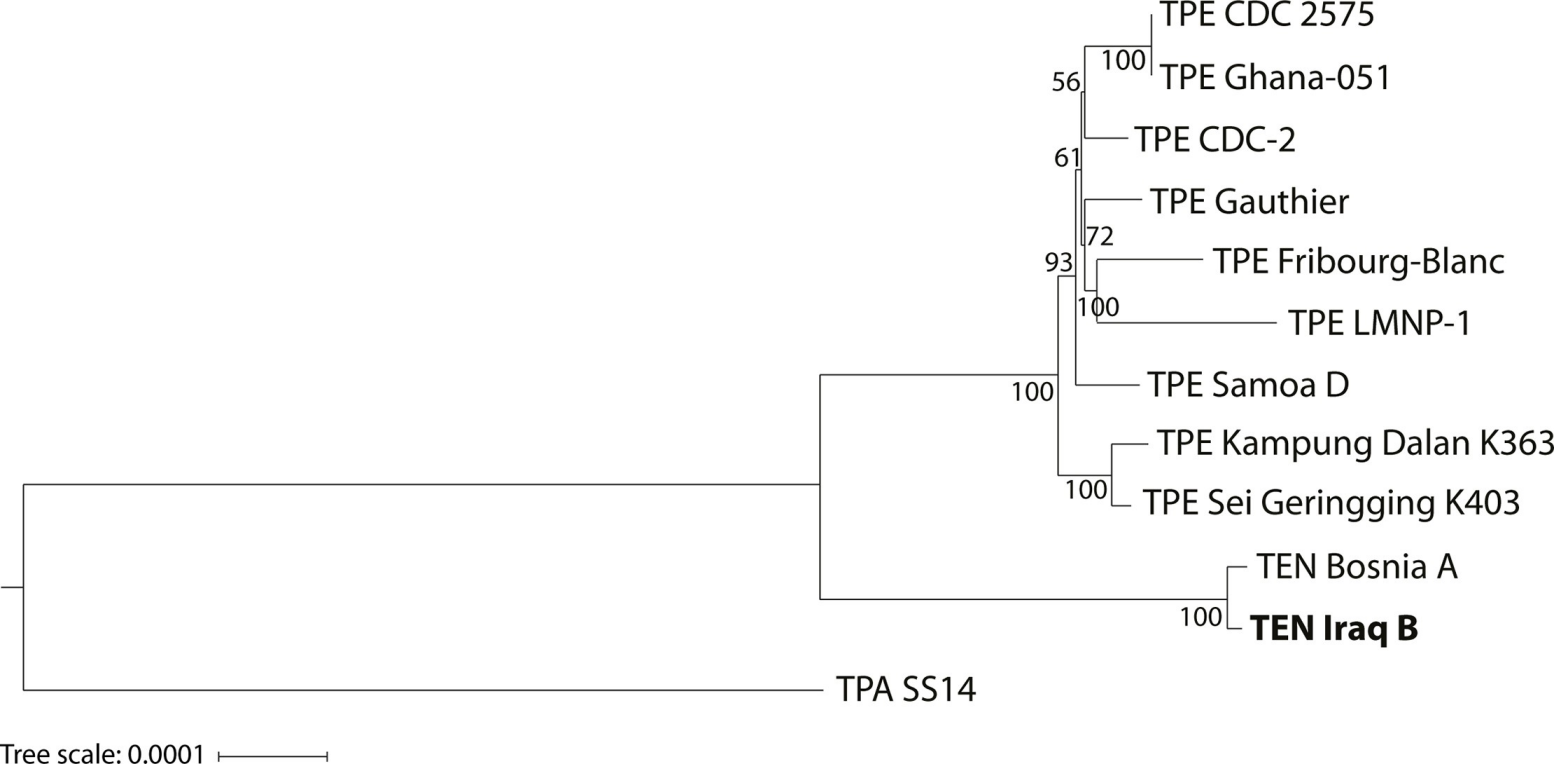
A phylogenetic tree based on the alignment of the *Treponema pallidum* subsp. *endemicum* (TEN) Iraq B genome with additional TEN and *T*. *pallidum* subsp. *pertenue* (TPE) genomes. The tree was constructed from the complete genome sequences of the TEN strains (Bosnia A, Iraq B) and TPE strains (CDC-2, Gauthier, Samoa D, Ghana-051, CDC 2575, Kampung Dalan K363, Sei Geringging K403, LMNP-1, and Fribourg-Blanc). The *tprK* sequences, tRNA-Ile and tRNA-Ala regions of both rRNA operons, *tprD*, *arp*, and TP0470 genes were omitted from the analysis due to their recombinant or repetitive character. The genome sequence of the *T*. *pallidum* subsp. *pallidum* (TPA) strain SS14 was used as an outgroup. There were a total of 1,129,405 positions in the final dataset. The Maximum Likelihood tree was constructed in RAxML-NG (v0.9.0) using the TN93 substitution model [23]. Gamma-distributed substitution rates among sites and proportion of invariable sites were applied. The robustness of the tree branches was assessed by 500-bootstrap replicate analyses. Predicted tree was visualized by iTOL (v5.5) [25]. The bar scale corresponds to a difference of 0.0001 nucleotides per nucleotide site.

The genome sequence of TEN Iraq B was most closely related to the genome of TEN Bosnia A and only distantly related to available TPE genomes (Fig 1). Despite the fact that only two TEN genomes were available, the phylogenetic tree showed a clear separation of the agents causing the endemic treponematoses, yaws and bejel.

### Intrastrain heterogeneity in the TEN Iraq B genome

The TEN Iraq B genome was assessed for the presence of intrastrain heterogeneity [20,21,26,27], with a frequency cutoff for minor alleles of 8% or more [20,21]. Altogether, 12 such sites were identified in TEN Iraq B, and the list of heterogeneous sites is shown in Table 3.

**Table 3:** Intrastrain heterogeneity found in the TEN Iraq B genome.

TEN Iraq B (CP032303.1) coordinates	Reference allele	Alternative allele	Amino acid replacement	Average depth coverage (x)	Percentage of alternative allele (%)	Gene	Protein
14661	C	T	R/C	1841	19.0	*pheT* (TP0015)	phenylalanine—tRNA ligase, beta subunit
135229	G	T	P/E	4380	44.5	*tprC* (TP0117)	TprC
135228	G	C	P/E	4360	44.4	*tprC*	
135246	C	T	R/K	3573	34.7	*tprC*	
136528	C	T	E/K	4264	26.0	*tprC*	
136542	C	T	R/H	3961	24.8	*tprC*	
334466	G	A	A/T	6111	8.1	*tmpC* (TP0319)	sugar ABC superfamily ATP binding cassette transporter
384160	G	T	A/S	3050	10.0	*cheA* (TP0363)	sensor histidine kinase
388371	G	A	D/N	2222	12.2	*cheY* (TP0366)	response regulator
824846	G	A	A/A	3614	12.7	(TP0762)	hypothetical protein
883419	G	T	G/W, P/H	2479	9.2	(TP0814a, TP0814b)	hypothetical proteins
			G/V			(TP0815)	GNAT family acetyltransferase
1091347	G	C	A/P	1005	9.3	(TP1003)	hypothetical protein

Minor alleles with a frequency over 8% and depth coverage over 100x are shown. The *tprK* (TP0897) gene was excluded from the analysis.

### Intrastrain heterogeneity in the length of G/C-homopolymeric tracts in TEN Iraq B

Intrastrain variability in the length of G/C-homopolymeric tracts was found at 18 positions in the TEN Iraq B genome (S2 Table). Although most of this variability was localized in intergenic regions, some occurred in four genes (TENIB_0461, TENIB_0479, TENIB_0865, and TENIB_1031) annotated as pseudogenes in the TEN Iraq B genome (S2 Table).

### Large insertion in the TEN Iraq B genome encoded by a minor treponemal population

**Fig 2:**
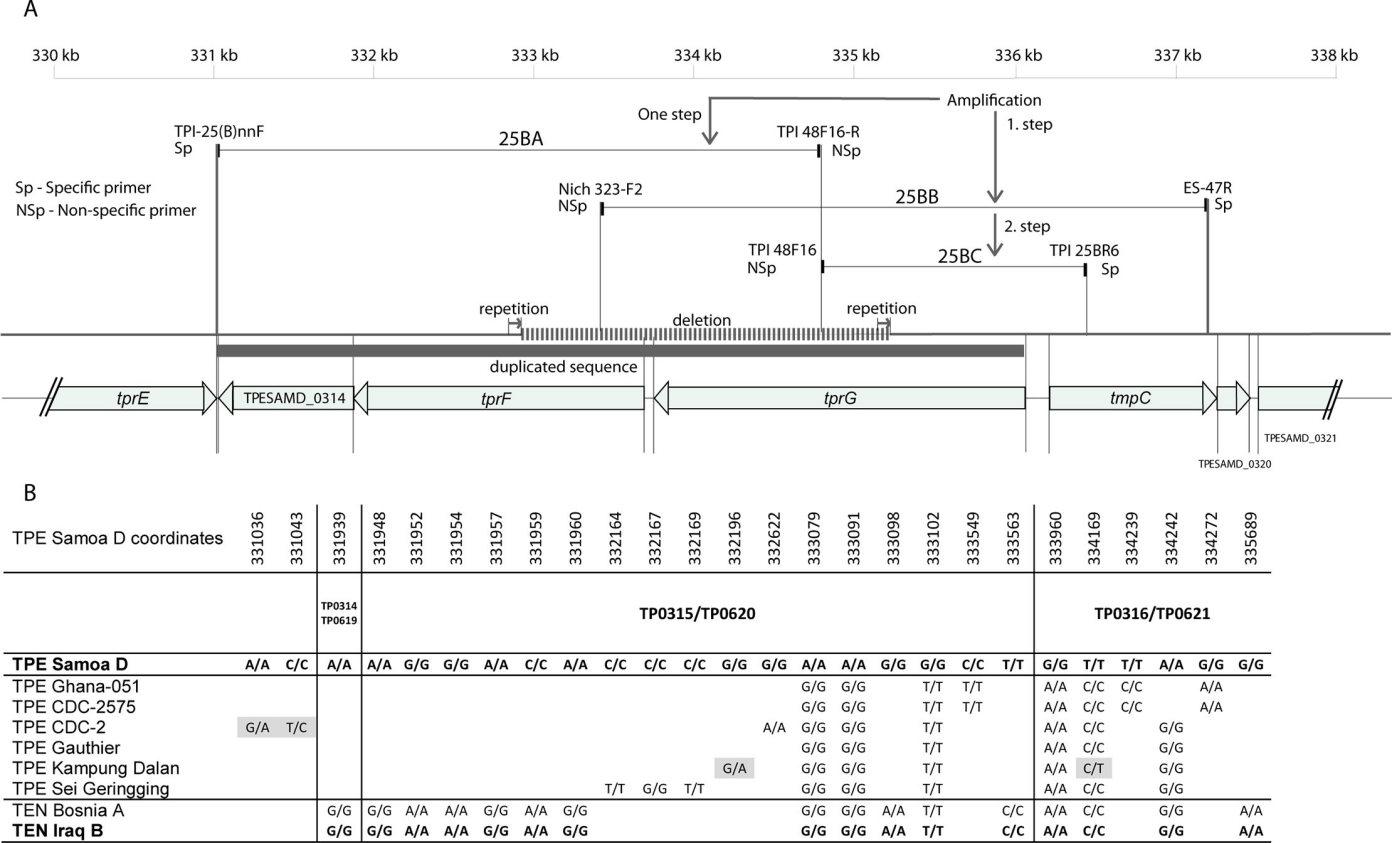
**A.** A schematic representation of a chromosomal region of the *Treponema pallidum* subsp. *endemicum* (TEN) Iraq B containing the TP0314-TP0318 genes. The entire genome region shown was amplified as two overlapping regions (25BA, 25BB), and each of these two loci contained one specific (Sp) and one non-specific (NSp) primer. Specific primers recognized unique sequences in the TEN Iraq B genome, while non-specific primers recognized binding sites in the paralogous TP0619-TP0621 region. The 25BA region was amplified in a one-step PCR, while the 25BC region was amplified in a second step with the template 25BB DNA (the 25BB template DNA did not have sufficient concentration for sequencing). The use of specific primers verified the amplification from the correct genome part. A 2.3 kb-long deletion present in a portion of the TEN population is shown and covers considerable parts of the *tprFG* loci (TP0316, TP0317). The paralogous sequence covering TP0314-TP0316 is also shown (see Fig 2B), and this sequence is identical to the region containing the *tprIJ* loci (TP0619-TP0621). Direct 75-bp long repetitions were found in the DNA regions flanking the deletion. **B.** A schematic representation of a paralogous sequence covering the TP0314-TP0316 and TP0619-TP0621 regions among available sequences from TEN and *T*. *pallidum* subsp. *pertenue* (TPE) genomes. Whereas the regions covering TP0314-TP0316 and TP0619-TP0621 are identical (or nearly identical) within a single TEN or TPE genome, intragenomic sequences of these regions are different among *T*. *pallidum* subsp. *pallidum*(TPA) strains [28]. Whereas individual TPE strains differed in 5–8 nucleotides from the reference sequence of TPE Samoa D within TP0314-TP0316 and TP0619-TP0621, TEN strains differed from TPE Samoa D in 15 nt positions. Two TPE strains (TPE Kampung Dalan, TPE CDC-2) did not have identical TP0314-TP0316 and TP0619-TP0621 loci and differed between them in 2 and 2 nucleotides, respectively. Some of the nucleotide differences were shared between both TEN and some TPE strains.

As described previously [2,28], the TEN Bosnia A genome contained a 2.3 kb-long deletion comprising the *tprF* and *tprG* loci (TP0316, TP0317) and the predicted TENDBA_0316 gene (1,8 kb) is a chimera previously described as *tprGI* [28]. A similar deletion was found in the TEN Iraq B genome [28]. Interestingly, amplification of the corresponding region in the TEN Iraq B genome revealed two bands, one corresponding to the version in the Bosnia A and one to the 2.3 kb-longer version present in the TPE genomes. Although the shorter, deleted version was predominantly amplified and represented the version present in the majority of Iraq B treponemes, we were able to specifically amplify and sequence the minor, non-deleted version (see S1 File). A schematic representation of this region is shown in Fig 2.

## Discussion

Here we describe the genome of Iraq B, the second complete genome sequence of the bejel-causing agent, *T*. *pallidum* subsp. *endemicum* (TEN). The genome was assembled using the previously described PSGS technique [2,15–17,20,21] and Illumina sequencing. The average coverage depth was greater than 3000× and all sequencing ambiguities were resolved with Sanger sequencing of the corresponding PCR products. However, the average coverage determination is slightly affected by the fact that DNA was first randomly and then specifically amplified even before the Illumina sequencing. Each detected difference from the Bosnia A genome was manually inspected in the original sequencing data from the strains TEN Bosnia A and Iraq B. It is therefore likely that the genome sequence contains minimal sequencing errors, if any.

The TEN Bosnia A and Iraq B strain genomes were highly related to each other and differed at only 37 single nucleotide variants (SNVs). However, in addition to SNVs, both genomes differed in the number of repetitions in the *arp* (TP0433; the number of repetitions matched the originally described numbers, [31]) and TP0470 genes, and in 4 indels (covering 7 nucleotides) found in a short homopolymeric region or in regions containing 2-nt long repetitions. In addition, differences between the genomes were found in the length of 18 homopolymeric tracts, defined as tracts having 8 or more identical nucleotides. All these simple sequence repeats (SSRs) are hypermutable, with a mutation rate in other bacteria about 10^−4^ per microbial generation [32]. Consistent with this, treponemal genomes expand or reduce the number of tandem repetitions in a relatively short evolutionary period and, as a result, SSRs do not correspond to the evolutionary relatedness of treponemal strains and subspecies [3]. A similar rule also appears to apply for tandem repetitions in the *arp* and TP0470 genes. As shown by Mikalová et al. [33], the number of *arp* repetitions is variable in different samples from the same patient. By contrast, the nucleotide mutation rate of TPA and TPE treponemes has been estimated to be 2.8 x 10^−10^ and 4.1 x 10^−10^ per site per generation or lower [20,34], suggesting treponemal genome stability for a decade (or decades).

The TEN Iraq B sequence was also analyzed with respect to the presence of intrastrain heterogeneity and these sites (Table 3) were compared with the heterogeneous sites identified in TEN Bosnia A genome in study by Čejková et al. [26]. Altogether, 12 and 6 intrastrain heterogeneous sites were identified in TEN Iraq B (this study) and TEN Bosnia A [26] genomes, respectively, but none of them were identical. These data support the previous assumption that treponemal heterogeneous sites are mostly strain specific [26]. However, lower average genome coverage in Bosnia A genome (513x) and different minor allele frequency cutoff in study by Čejková et al. [26] could potentially affect the number of identified heterogeneous sites. Since both TEN strains Bosnia A and Iraq B were cultivated in rabbits, it is unknown whether the identified genetic variability resulted from serial passages of these strains in rabbits or was already present during infection of humans.

The length of the Iraq B strain’s genome is identical to that of the Bosnia A strain (1,137,653 bp). Both genomes are thus about 2-kbp shorter than the length of other TPE or TPA genomes analyzed to date [1,3]. The reason for this is a 2.3 kbp deletion in the *tprF* and *tprG* loci, originally found by Centurion-Lara et al. [28]. However, an identical deletion spanning *tprF* and *tprG* loci was also found in the *T*. *paraluiscuniculi* strain Cuniculi A [35,36], later renamed *T*. *paraluisleporidarum* ec. Cuniculus, strain Cuniculi A [37]. In previous work by Štaudová et al. [2], this 2.3 kb deletion was proposed to result from polymerase slippage between the repeats in the flanking regions of the deleted 2.3-kb region, which could have happened repeatedly during evolution. This deletion was also observed during experimental amplification of the *tprFG* loci in additional genomes containing full versions of this chromosomal region [2].

Interestingly, during the PSGS amplification of the region covering this deletion in the TEN Iraq B genome, bands corresponding to both the deleted and undeleted versions of *tprFG* were identified. The larger version revealed a sequence related to (but genetically distinct from) the sequences present in TPE treponemes, suggesting that a portion of the population of TEN Iraq B treponemes carry the undeleted version. Since the *tprFG* region is 635 bp shorter in TPA strains [38] than in TPE strains, this minority of TEN microbes resemble TPE strains in this region, a feature typical of the entire TEN genome (Fig 1). In addition, in all TPE and TEN strains analyzed to date, a paralogous sequence covering TP0314-TP0316 is identical (or nearly identical) to the region containing the *tprIJ* loci (TP0619-TP0621) (Fig 2B). The duplicate character of the TP0314-TP0316 and TP0619-TP0621 regions could explain why this relatively large deletion (2.3 kb) does not represent a major fitness cost that would lead to elimination of the treponemes with deleted *tprFG* genes. Instead, this deletion appears to be in a process of fixation in the TEN genomes. No such deletion comprising *tprFG* genes was found among TPE strains, despite the same duplicate character of the TP0314-TP0316 and TP0619-TP0621 regions and despite the presence of direct repeats.

A potential contamination of TEN Iraq B DNA with the DNA from TPE microbes (that could result in a similar finding of full-length versions of *tprFG* loci) can be excluded from several reasons including i) absence of intrastrain heterogeneity in sites differentiating TEN and TPE strains, ii) absence of alternative indels (e.g., in *tprL*) and iii) sequence differences between the TEN *tprFG* and TPE *tprFG* loci (Fig 2B).

The observed deletion in the TEN genomes is one of the largest detected genetic changes found among *Treponema pallidum* strains; however, a deletion of similar size (1,204 bp in length) was found in 6 out of 21 clones harboring TP0126 during the preparation of the large insert BAC library from the DNA of the Nichols strain [39]. This 1,204 bp region contained a *tprK*-like sequence in the intergenic region between the TP0126 and TP0127 genes, and a similar sequence was found also in the DAL-1 genome [38,40]. Among SS14-like strains, including SS14 and the Mexico A strain [41,42], this sequence was slightly longer (1,255 bp) [38]. Both these examples demonstrate that substantial deletions of the treponemal chromosome can occur and that deletion in a part of the population could eventually lead to loss of the chromosomal region in the entire population. Another example of a genome containing multiple deletions involves the chromosome of *T*. *paraluisleporidarum* ec. Cuniculus [35,36].

All pathogenic treponemal strains causing human infections have sequence identity greater than 99.7% [3]. The high genetic relatedness of TPA, TPE, and TEN strains explains why the etiological agents of syphilis, yaws, and bejel cannot be distinguished morphologically and induce indistinguishable serological responses. Moreover, clinical symptoms of these diseases may overlap, especially in the early stages of disease, and differential diagnosis is made largely on anamnestic data and epidemiological context. It has been shown that in very dry areas, yaws and bejel symptoms are highly overlapping [43]. The clinical manifestation of TPA and TEN infections is also highly similar, and recent reports have described bejel treponemes in patients suspected of having syphilis in France, Cuba, and Japan [9–12]. Despite the relatedness of the TPA, TPE, and TEN treponemes, strains belonging to different subspecies cluster together, and this clustering corresponds to disease classification. It is therefore tempting to speculate that the genetic differences between the TEN and TPA genomes code for differences in disease manifestations and perhaps geographical occurrence.

The most prominent differences between TPA and TEN genomes were observed in the family of *tpr* genes. Many of the Tpr proteins are predicted outer membrane proteins [30] and induce an antibody response during infection [44–46]. It is therefore believed that Tpr proteins are of importance in treponemal pathogenicity and in the pathogen’s interplay with the host immune system. The *tprA* gene was intact in both the TEN Iraq B and Bosnia A genomes, similar to TPE strains. This is different from TPA strains, in which the *tprA* gene is annotated as a pseudogene, with the exception of TPA strain Sea 81–4, which appears to have a functional *tprA* gene [47]. All *tprB*, *tprC*, *tprD*, and *tprE* gene loci confer functional alleles among human TPA, TPE, and TEN strains, with the *tprD2* allele in the *tprD* locus of TEN strains. *tprFG* was found partially deleted in TEN genomes (similarly to *T*. *p*. ec. Cuniculus genome), the *tprF* gene among TPA strains appears to be a pseudogene, and the *tprF* gene among TPE strains appears to encode a full-length, functional protein. The *tprG* gene, partially deleted in TEN strains (*tprGI* chimera; [28]), is functional in TPA (truncated in Sea 81–4; [47]) and TPE (*tprGJ* chimera; [28]) strains. The *tprL* gene in the TEN strains was longer than in TPE genomes (378 bp-longer; [2]), similar to what is seen in TPA strains [28]. Interestingly, *tprL* was identified as a pseudogene in Iraq B, due to expansion of a homopolymeric (G) region (S2 Table). All *tprH*, *tprI*, *tprJ*, and *tprK* gene loci confer functional alleles among TPA, TPE, and TEN strains. *tprK* gene that was previously found to be highly variable within and among treponemal strains [48], revealed also genetic variability in TEN Iraq B with the diversity accumulated mostly in seven discrete variable regions [45,49,50]. A gene conversion mechanism was proposed to be responsible for generating the heterogeneity within the *tprK* gene [49] with donor sites localized in flanking regions of the *tprD* gene [51]. Differences between TPA and TPE/TEN in the *tprK* donor sequences in the *tprD* flanking regions exist [51]. The diversity of TprK accumulates during infection enabling the pathogen to escape the host immune response and allowing the pathogen to persist in the host [50,52]. The involvement of TprK in immune evasion was experimentally shown by Giacani et al. [53] and Reid et al. [54]. Moreover, recent studies by Pinto et al. [27] and Liu et al. [55,56] used next-generation sequencing to characterize the diversity of *tprK* directly from syphilis patients and revealed differences in profiles of *tprK* between primary and secondary syphilis [56]. Taken together, the *tpr* genes appear to show unique patterns in TPA, TPE, and TEN strains.

In sum, in this work we present a complete, high-quality genome sequence of the TEN lab strain Iraq B, the second TEN strain sequenced to date alongside Bosnia A. As shown in this study, a subpopulation of TEN Iraq B treponemes still contain the *tprFG* loci that was previously described as deleted in the TEN Bosnia A genome. Analysis of the TEN Iraq B genome revealed an ongoing process of deletion of chromosomal regions during the evolution of these pathogenic treponemes. Moreover, strains of the TPA, TPE, and TEN treponemes clustered separately, indicating that this clustering corresponds to disease and subspecies classifications.

## Conclusions

A high-quality complete genome sequence for TEN Iraq B revealed close genetic relatedness between both available bejel-causing lab strains (i.e., Iraq B and Bosnia A) and also genetic variability within the bejel treponemes comparable to that found within yaws- or syphilis-causing strains. In addition, genetic relatedness to TPE strains was demonstrated by the sequence of the *tprF* and *tprG* genes found in subpopulations of both TEN Iraq B and Bosnia A. The loss of the *tprF* and *tprG* genes in most TEN microbes represents an adaptive evolutionary process and suggests that TEN genomes have been evolving via the loss of genomic regions, a phenomenon previously found among the treponemes causing both syphilis and rabbit syphilis.

## Supporting information

S1 Table PSGS (Pool Segment Genome Sequencing) approach. List of treponeme-specific primers used for the amplification of TP intervals of TEN Iraq B strain.
(XLSX)

S2 Table Genetic differences between TEN Bosnia A and Iraq B genomes in the length of 18 homopolymeric tracts. (DOCX)

S1 File The electrophoretogram of 25BA and 25BC regions of the TEN Iraq B. The PCR products showing the undeleted version of *tprFG* region are shown. The 25BA region was amplified by primers TPI-25(B)nnF (5'-GGGCGCCTTCCGACAGGACCCG-3') and TPI48F16-R (5'-GGTGGTGAAGGGGTTTGAGC-3') in a one-step PCR while the 25BC region was amplified by primers TPI48F16 (5'-GCTCAAACCCCTTCACCACC-3') and TPI25BR6 (5'-GACAAACTAGGCACGTACTC-3') in a second step with the template 25BB DNA (the 25BB template DNA did not have sufficient concentration for sequencing). A schematic representation of a chromosomal region of the TEN Iraq B containing the *tprFG* region is shown in Fig 2.
(PDF)
