# Supplementary material for: Whole genome sequence of the Treponema pallidum subsp. endemicum strain Iraq B: A subpopulation of bejel treponemes contains full-length tprF and tprG genes similar to those present in T. p. subsp. pertenue strains
Source: PLoS One. 2020 Apr 1;15(4):e0230926. doi: 10.1371/journal.pone.0230926 (PMC7112178; doi:10.1371/journal.pone.0230926)
Supplement: S2 Table — (DOCX) [file pone.0230926.s002.docx]

**S2 Table. Genetic differences between TEN Bosnia A and Iraq B genomes in the length of 18 homopolymeric tracts.**

| **TEN Iraq B (CP032303.1.) coordinates** | **Nucleotide in TEN Iraq B** | **Nucleotide in TEN Bosnia A** | **Gene** | **Protein/remark** |
| --- | --- | --- | --- | --- |
| 122202-209 | 8C | 9C | IGR TP0007-8* |  |
| 136708-716 | 9C | 10C | IGR TP0117-8 |  |
| 140921-928 | 8C | 9C | IGR TP0121-2 |  |
| 154144-152 | 9C | 10C | IGR TP0131-3** |  |
| 199629-638 | 10G | 11G | IGR TP0179-81*** |  |
| 208538-545 | 8G | 9G | IGR TP0192-3 |  |
| 371454-463 | 10G | 13G | TP0347 | hypothetical protein |
| 404619-629 | 11C | 10C | IGR TP0379-80 |  |
| 407350-365 | 16C | 13C | IGR TP0381-3**** |  |
| 490396-402 | 7C | 8C | TP0461 | putative transcriptional regulator, frameshift mutation |
|  |  |  | TP0461a***** | hypothetical protein |
| 490424-432 | 9G | 8G | TP0461 | putative transcriptional regulator, frameshift mutation |
|  |  |  | TP0461a***** | hypothetical protein |
| 508990-999 | 10C | 9C | TP0479 | hypothetical protein, frameshift mutation |
| 674803-812 | 10C | 9C | IGR TP0621-2 |  |
| 935982-991 | 10G | 13G | TP0859 | FadL-like protein****** |
| 943663-671 | 9C | 11C | TP0865 | FadL-like protein******, frameshift mutation |
| 1004988-996 | 9C | 8C | IGR TP0924-t0042 |  |
| 1048789-798 | 10C | 8C | IGR TP0966-7 |  |
| 1123689-700 | 12G | 10G | TP1031 | TprL, frameshift mutation |

Homopolymeric tracts were defined as stretches of identical nucleotide sequences longer that 7 nucleotides. *IGR, intergenic region.

**TP0132 is not annotated in TEN.

***TP0180 is not annotated in TEN

****TP0382 is not annotated in TEN.

*****Overlapping genes.

******Protein predictions by Radolf and Kumar (2018).

Radolf JD, Kumar S. The *Treponema pallidum* outer membrane. Curr Top Microbiol Immunol. 2018;415: 1-38.
