# Supplementary material for: Whole genome sequence of the Treponema pallidum subsp. endemicum strain Iraq B: A subpopulation of bejel treponemes contains full-length tprF and tprG genes similar to those present in T. p. subsp. pertenue strains
Source: PLoS One. 2020 Apr 1;15(4):e0230926. doi: 10.1371/journal.pone.0230926 (PMC7112178; doi:10.1371/journal.pone.0230926)

S3 File. The electrophoretogram of 25BA and 25BC regions of the TEN Iraq B. The PCR products showing the undeleted version of *tprFG* region are shown. The 25BA region was amplified by primers TPI-25(B)nnF (5'-GGGCGCCTTCCGACAGGACCCG-3') and TPI48F16-R (5'-GGTGGTGAAGGGGTTTGAGC-3') in a one-step PCR while the 25BC region was amplified by primers TPI48F16 (5'-GCTCAAACCCCTTCACCACC-3') and TPI25BR6 (5'-GACAAACTAGGCACGTACTC-3') in a second step with the template 25BB DNA (the 25BB template DNA did not have sufficient concentration for sequencing). A schematic representation of a chromosomal region of the TEN Iraq B containing the *tprFG* region is shown in Figure 2.

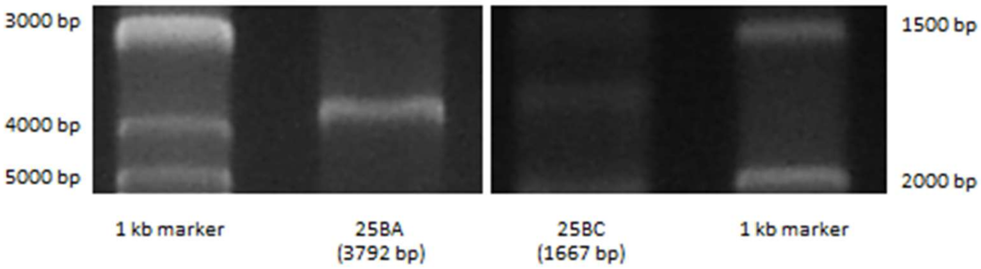

Supplement: S1 File — The PCR products showing the undeleted version of tprFG region are shown. The 25BA region was amplified by primers TPI-25(B)nnF (5'-GGGCGCCTTCCGACAGGACCCG-3') and TPI48F16-R (5'-GGTGGTGAAGGGGTTTGAGC-3') in a one-step PCR while the 25BC region was amplified by primers TPI48F16 (5'-GCTCAAACCCCTTCACCACC-3') and TPI25BR6 (5'-GACAAACTAGGCACGTACTC-3') in a second step with the template 25BB DNA (the 25BB template DNA did not have sufficient concentration for sequencing). A schematic representation of a chromosomal region of the TEN Iraq B containing the tprFG region is shown in Fig 2. (PDF) [file pone.0230926.s003.pdf]
